# KAR_1_-induced dormancy release in *Avena fatua* caryopses involves reduction of caryopsis sensitivity to ABA and ABA/GA_s_ ratio in coleorhiza and radicle

**DOI:** 10.1007/s00425-024-04387-1

**Published:** 2024-04-18

**Authors:** Jan Kępczyński, Michal Dziurka, Agata Wójcik

**Affiliations:** 1 Institute of Biology, University of Szczecin, Waska 13, 71-415 Szczecin, Poland; 2 Institute of Plant Physiology, Polish Academy of Sciences, Niezapominajek 21, 20-239 Krakow, Poland

**Keywords:** Abscisic acid, Coleorhiza, Dormancy, Gibberellins, Karrikin 1, Radicle, Wild oat

## Abstract

**Main conclusion:**

The dormancy release by KAR_1_ is associated with a reduction of coleorhiza and radicle sensitivity to ABA as well as with reduction the ABA/GA_s_ ratio in the coleorhiza, by a decrease content of ABA, and in the radicle, by a decrease the ABA and an increase of the GA_s_ contents.

**Abstract:**

Both, karrikin 1 (KAR_1_) and gibberellin A_3_ (GA_3_), release dormancy in *Avena fatua* caryopses, resulting in the emergence of coleorhiza (CE) and radicle (RE). Moreover, KAR_1_ and GA_3_ stimulate CE and RE in the presence of abscisic acid (ABA), the stimulation being more effective in CE. The stimulatory effects of KAR_1_ and GA_3_ involve also the CE and RE rates. A similar effect was observed at KAR_1_ concentrations much lower than those of GA_3_. KAR_1_ increased the levels of bioactive GA_5_ and GA_6_ in embryos and the levels of GA_1_, GA_5_, GA_3,_ GA_6_ and GA_4_ in radicles. The stimulatory effect of KAR_1_ on germination, associated with increased levels of gibberellins (GA_s_) and reduced levels of ABA in embryos, was counteracted by paclobutrazol (PAC), commonly regarded as a GA_s_ biosynthesis inhibitor. Consequently, KAR_1_ decreased the ABA/GA_s_ ratio, whereas PAC, used alone or in combination with KAR_1_, increased it. The ABA/GA_s_ ratio was reduced by KAR_1_ in both coleorhiza and radicle, the effect being stronger in the latter. We present the first evidence that KAR_1_-induced dormancy release requires a decreased ABA/GA_s_ ratio in coleorhiza and radicle. It is concluded that the dormancy-releasing effect of KAR_1_ in *A. fatua* caryopses includes (i) a reduction of the coleorhiza and radicle sensitivity to ABA, and (2) a reduction of the ABA/GA_s_ ratio (i) in the coleorhiza, by decreasing the ABA content, and (ii) in the radicle, by decreasing the ABA and increasing the content GA_s_, particularly GA_1_. The results may suggest different mechanisms of dormancy release by KAR_1_ in monocot and dicot seeds.

## Introduction

**Fig. 1:**
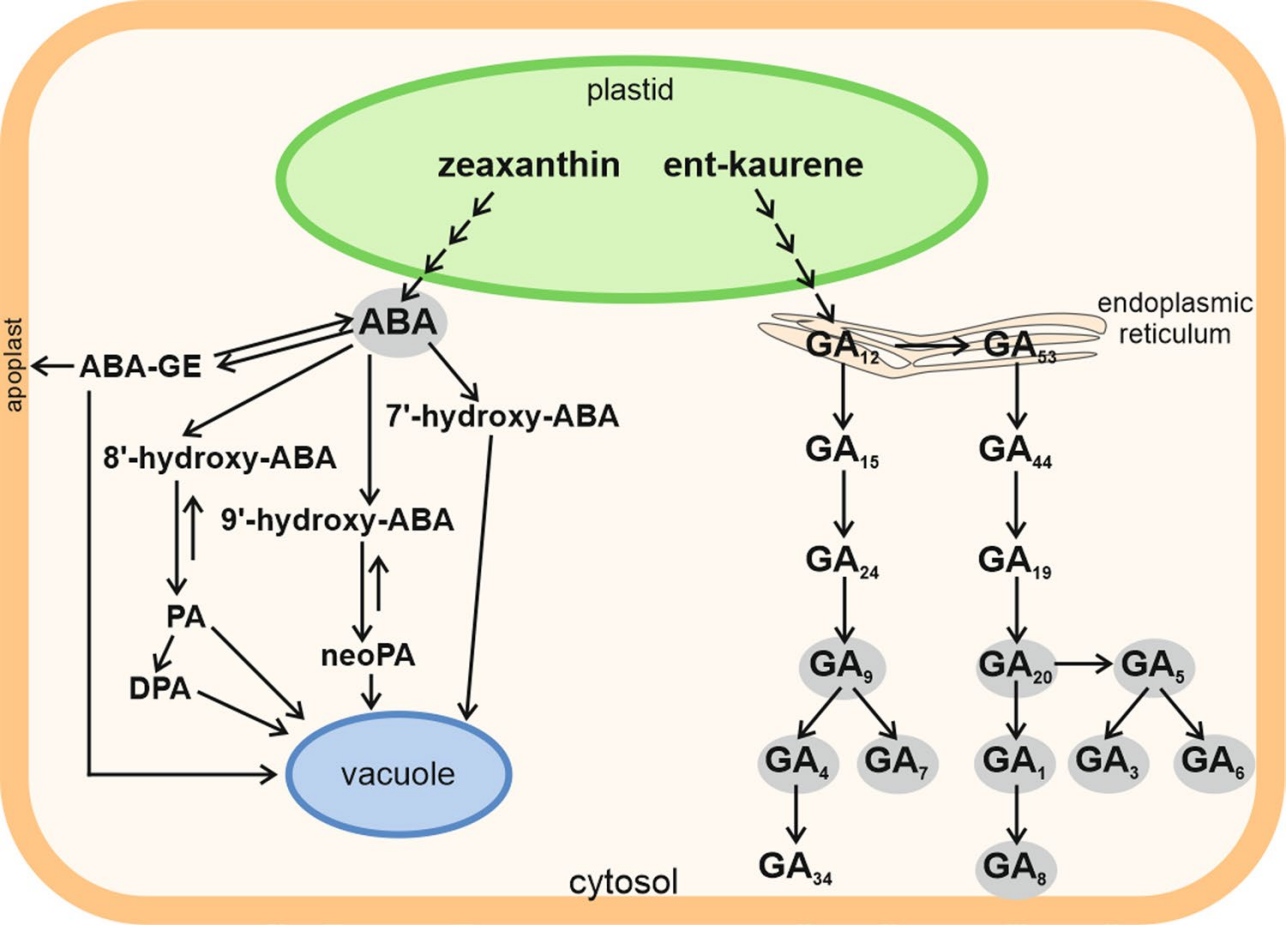
Diagram illustrating the metabolic pathways of ABA and GA_s_. The compounds analyzed in this work are shown as grey fields. Adapted from Kępczyńska and Orlowska (2021)

Harvested intact viable seeds of many plant species from both monocotyledonous and dicot plants are not able to complete germination under suitable species-specific conditions (water, temperature, air, light). Such seeds are regarded as primarily dormant (Bewley 2013). Primary dormancy established during seed maturation is very important in the wild plant life, as it facilitates survival and dispersal of the species. On the other hand, seed dormancy in agricultural crop weeds makes their control difficult. It is widely accepted that the balance between ABA and GA_s_, which are synthesized in cytosol (Fig. 1), is responsible for the state of seed dormancy. ABA plays a key role in the induction and maintenance of dormancy, and GA_s_ participate in dormancy release and/or germination (Finkelstein et al. 2008). *Avena fatua* is an example of an annual weed grass which infests major cereals in the world, including Poland, and produces primarily dormant caryopses which show physiological dormancy. This dormancy is expressed as the absence of complete germination at warmer temperatures at which non-dormant caryopses can germinate. Dormant caryopses of the grass in question, able to remain viable in a soil bank for several years, have been used as a model material in the study of the dormancy release mechanism in monocots (Simpson 1990; Kępczyński 2018, 2023).

Dormancy of *A. fatua* caryopses/florets can be removed by various treatments. It is released by after-ripening during dry storage of florets (Kępczyński et al. 2021) as well as by treatments involving GA_3_ (Kępczyński 2018) or nitric oxide (NO) (Kępczyński et al. 2021). The NO treatment was observed to be ineffective with respect to the content of GA_s_ from non-13-hydroxylation and 13-hydroxylation pathways considered as bioactive (Hedden and Phillips 2000), but strongly decreased the content of ABA (Kępczyński et al. 2021). In addition, although the dormancy-releasing effect of NO was counteracted by paclobutrazol (PAC), a GA_s_ biosynthesis inhibitor which blocks the GA_s_ biosynthesis by inhibiting ent-kaurene oxidation (Desta and Amare 2021), PAC did not affect the GA_s_ contents, however it increased the ABA content (Kępczyński et al. 2021). The dormancy release by NO has been concluded to involve a decrease in the ABA/GA_s_ ratios and a reduction of caryopsis sensitivity to ABA. Other factors such as plant-derived smoke, smoke water and KAR_1_ isolated from smoke are also known to stimulate seed germination in numerous species, including dormant caryopses of *A*. *fatua* (Kępczyński 2023). KAR_1_ has been found to be more effective in releasing dormancy than GA_3_. Interaction between KAR_1_ and PAC suggested that induction of germination in dormant *A. fatua* caryopses involves endogenous GA_s_ (Ruduś et al. 2019). As shown by Kępczyński and Van Staden (2012), although exogenous ethylene did not release caryopsis dormancy completely, to remove dormancy due to KAR_1_ endogenous ethylene action was required. Also, as reported by Ruduś et al. (2019), the effect of KAR_1_ was associated with non-transcriptional and transcriptional activation of ACC synthase and ACC oxidase and with modulation of the sensitivity to ethylene by regulation synthesis of ethylene receptors.

It is known that, following dormancy release, the sensu stricto germination of both monocot and dicot non-dormant seeds has been completed when the radicle or other embryonic tissue emerges through the structure covering it (Bewley et al. 2013). In the case of caryopses of, e.g., barley, *Brachypodium distachyon* and *A. fatua*, the radicle is sheathed by coleorhiza and germination is considered to involve two stages (González-Calle et al. 2015; Holloway et al. 2020; Kępczyński et al. 2021). During the first stage, the coleorhiza breaks through the surrounding structures, the second stage being associated with radicle emergence through the coleorhiza. Therefore, the sensu stricto germination is completed when the coleorhiza in monocots is punctured by radicle. So far, studies involving caryopses have used different germination criteria: the coleorhiza emergence from the surrounding tissues (Gubler et al. 2008; Kępczyński 2023), the radicle emergence through the coleorhiza (Gendreau et al. 2008) or both criteria simultaneously (Jacobsen et al. 2013; González-Calle et al. 2015; Holloway et al. 2020; Kępczyński et al. 2021). Previously, the coleorhiza was considered as responsible for protecting the emerging radicle (Sargent and Osborne 1980), whereas at present it is recognized as playing also a key role in grass caryopsis dormancy (Millar et al. 2006; Barrero et al. 2009), including in *A. fatua* (Holloway et al. 2020). Moreover, it was postulated that ABA reduction in the coleorhiza is very important in controlling caryopsis dormancy and germination of barley (Millar et al. 2006; Barrero et al. 2009) and *A. fatua* (Kępczyński et al. 2021).

The present study was aimed at highlighting the relationship between KAR_1_ and gibberellins (GA_s_) as well as ABA in dormancy release in *A. fatua* caryopses. This was done by studying the effect of KAR_1_ and GA_3_ on the final percentage and speed of the dormant caryopses’ coleorhiza and radicle emergence in the absence or in the presence of ABA. The relationship between KAR_1_ and endogenous GA_s_ was investigated by determining contents of bioactive GA_s_ from the non-13-hydroxylation pathway (GA_4_ and GA_7_) and from the 13-hydroxylation pathway (GA_1_, GA_5_, GA_3_ and GA_6_) in embryos from caryopses germinated in the presence of KAR_1_. In addition, effects of PAC, KAR_1_ and PAC + KAR_1_ on the GA_s_ and ABA contents were explored. Moreover, the GA_s_ level was determined in the coleorhiza and radicle of embryos isolated from caryopses treated with KAR_1_. Further, changes in the ABA/GA_s_ ratio in embryos, coleorhiza and radicle in KAR_1_-treated caryopses were investigated. The results provide new information on the role of KAR_1_ in releasing caryopsis dormancy of grasses in relation to ABA/GA_s_ ratios in the coleorhiza and radicle before germination is completed.

## Material and methods

*Avena f*atua L. spikelets, containing florets, collected in 2015, were dried to a constant moisture content (ca. 11%) and stored at – 20 ℃ until used.

### Germination assays

**Fig. 2:**
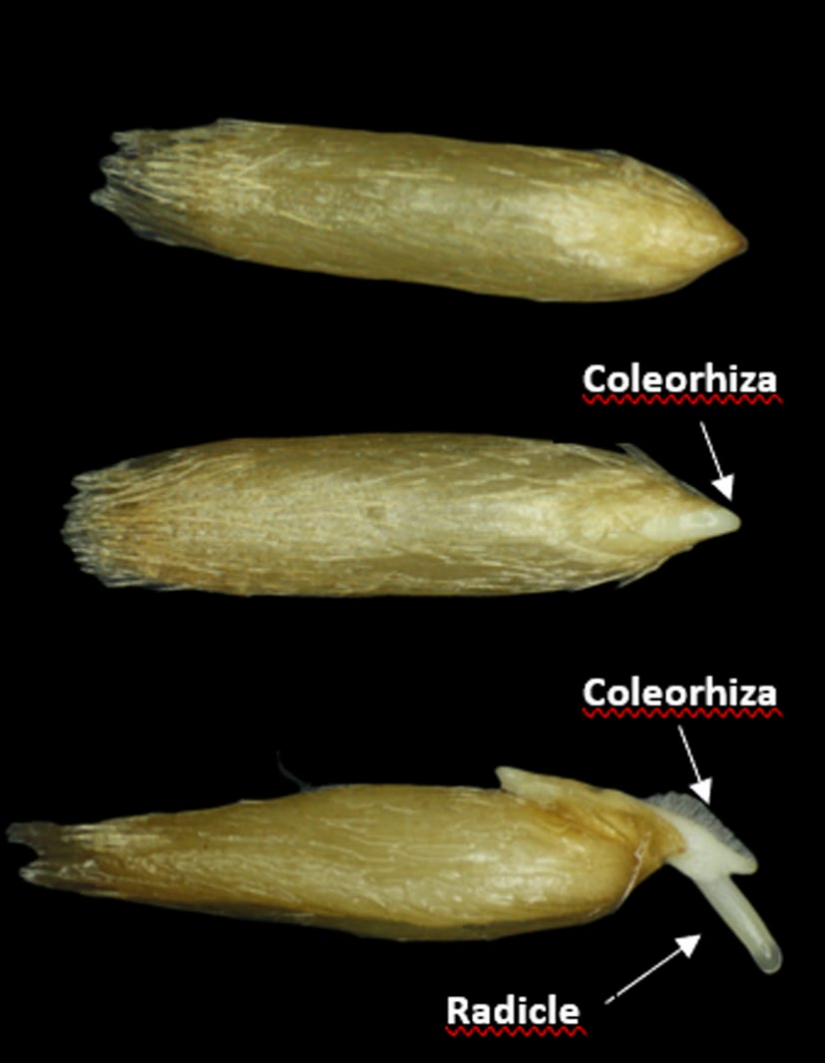
Germination of *A. fatua* caryopses. Photograph show ungerminated caryopsis, caryopsis with emerged coleorhiza and caryopsis with emerged radicle (germinated caryopsis)

Air-dried dormant caryopses (florets without the lemma and palea) (25 in 3 replicates) were incubated in the dark at 20 °C in Petri dishes (6 cm diameter) on a single layer of filter paper (Whatman No.1) moistened with 1.5 ml distilled water, KAR_1_ (10^–9^, 3 × 10^–9^,10^–8^ M), GA_3_ (10^–5^, 10^–4^, 10^–3^ M), ABA (10^–5^, 3 × 10^–5^, 10^–3^ M), KAR_1_ + ABA, GA_3_ + ABA, PAC (10^–4^ M),, and KAR_1_ (3 × 10^–9^ M) + PAC. Chemicals, except for PAC which was dissolved in acetone, were dissolved in water at room temperature (KAR_1_) or in water heated to ca. 40 ℃. Caryopses with CE over the coat and with RE through the coleorhiza were counted every day until day 7 of germination (Fig. 2). All manipulations were performed under green light which did not affect germination. Effects of the compounds used on dormancy release were characterized by the final percentage of caryopses and Timson´s index (Σ_7_), calculated by summing up the CE or RE percentages over 7 days (Timson 1965).

### Determination of GA_s_ and ABA contents

Dormant caryopses (25 in 3 replicates) were incubated in the dark at 20 ℃ in Petri dishes (6 cm diameter) on a single layer of filter paper (Whatman No. 1) moistened with 1.5 ml distilled water, KAR_1_ (3 × 10^–9^ M) for 18, 24, 30 or 36 h or KAR_1_ + PAC (10^–4^ M) for 30 h. Upon completion of incubation, the embryos (after 18, 24 and 36 h) or coleorhizae and radicles (after 24 h) were isolated, and GA_s_ and ABA were analyzed as described previously (Kępczyński et al. 2021, 2023). In Fig. 4 and 5 the GA_s_ contents in embryos incubated in water or PAC, demonstrated previously, were used. Likewise, the data of ABA contents obtained in previous studies (Kępczyński et al. 2021, 2023) were used for calculating the ABA/GA_s_ ratios.

### Statistical treatment

The mean ± standard deviation (SD) of three replicates was calculated. Significance of differences between the means was tested using one- or two-way analysis of variance (ANOVA; Statistica for Windows v. 10.0, Stat-Soft Inc., Tulsa, OK, USA). Duncan’s multiple range test was used to identify significantly different (*P* ≤ 0.05) mean values.

## Results

### Effects of KAR_1_ and GA_3_ on the emergence of coleorhiza and radicles of ***A. fatua*** caryopses in the absence or in the presence of ABA

**Fig. 3:**
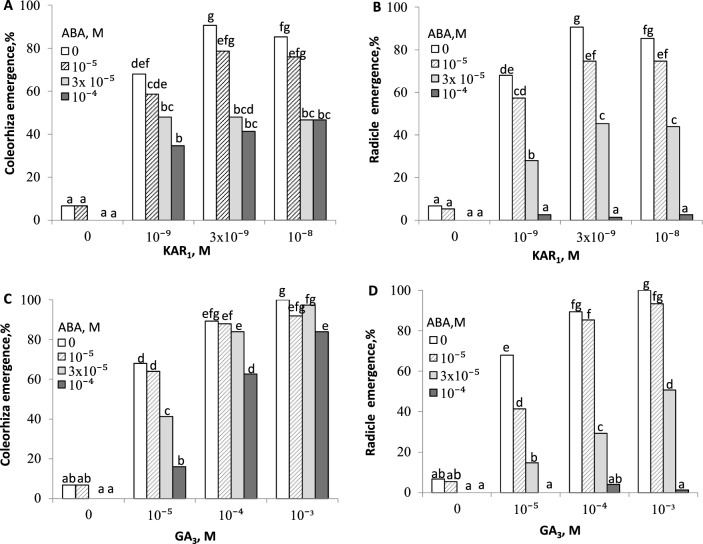
Effects of KAR_1_ and GA_3_ on the emergence of coleorhiza (**A**, **C**) and radicle (**B**, **D**) of *A. fatua* in the absence or in the presence of ABA after 7 days of germination. One way ANOVA with Duncan´s post hoc test was used to test for significance of differences. Means denoted by different letters differ significantly (*P* < 0.05, *n* = 3)

Both, coleorhizae and radicles, of the primarily dormant *A. fatua* caryopses incubated in water or ABA solutions were almost or totally unable to emerge (0–5%) (Fig. 3). KAR_1_ stimulated the coleorhiza emergence (CE) in all the concentrations used (Fig. 3A). The highest and similar levels of stimulation were found after KAR_1_ was applied at concentrations of 3 × 10^–9^ and 10^–8^ M; ca. 90% of caryopses observed show CE. KAR_1_ applied at concentrations of 10^–9^- 10^–8^ M produced a similar CE despite the presence of ABA at 10^–5^ M (ca. 80%). Application of KAR_1_ at 3 × 10^–9^ or 10^–8^ M markedly enhanced CE in the presence of 3 × 10^–5^ and 10^–4^ M ABA; ca. 50% of coleorhizae emerged. KAR_1_ used 3 × 10^–9^ and 10^–8^ M resulted in ca. 90% RE (Fig. 3B). The highest antagonizing KAR_1_ effect was found when ABA was used at the lowest concentration (10^–5^ M). KAR_1_ at all concentrations did not affect germination in the presence of ABA at the highest concentration of 10^–4^ M (ca. 3%). KAR_1_ at 10 ^−8^ M increased Timson´s index (Σ_7_) by the factor of 21 and 23 in the coleorhiza and radicle, respectively (Table 1A). In the presence of ABA at 10^–5^ M, KAR_1_ at 10 ^−8^ M did enhance Timson´s index in both the coleorhiza and radicle by the factor of 39 and 31, respectively.

**Table 1:** Effects of KAR_1_ (A) and GA_3_ (B) on the speed (Timson´s index) of coleorhiza and radicle emergence in *A. fatua* in the presence of ABA

	ABA, M			
	Coleorhiza		Radicle	
	0	10^–5^	0	10^–5^
(A) KAR_1_, M				
0	21.3 ± 20.1a	10.7 ± 12.2a	17.3 ± 16.2a	10.7 ± 12.2a
10^–9^	373.3 ± 56.6d	289.3 ± 35.9b	336.0 ± 52.9c	216.0 ± 32.7b
10^–8^	448.0 ± 32.7d	414.7 ± 46.0 cd	405.3 ± 38.0d	337.3 ± 23.1c
(B) GA_3_, M				
0	21.3 ± 20.1a	10.7 ± 12.2a	17.3 ± 16.2a	10.7 ± 12.2a
10^–5^	381.3 ± 44.2c	244.0 ± 46.1b	353.3 ± 28.1d	121.3 ± 15.1b
10^–4^	500.0 ± 41.8d	360.0 ± 32.0c	465.3 ± 47.7e	216.0 ± 38.2c

One way ANOVA with Duncan´s post hoc test was used to test for significance of differences. Means denoted by different letters differ significantly (*P* < 0.05, *n* = 3)

Likewise, GA_3_ stimulated CE and RE at all the concentrations used (Fig. 3C, D). The highest stimulatory effect on CE and RE of dormant caryopses was observed at GA_3_ concentrations of 10^–4^ and 10^–3^ M; almost all the caryopses (90–100%) showed CE and RE. When used at the concentrations mentioned, GA_3_ resulted in 90% of the caryopses showing CE, despite the presence of ABA at 10^–5^- 3 × 10^–5^ M (Fig. 3C). When used at the two highest concentrations in the presence of 10^–5^ M ABA, GA_3_ produced an almost complete RE (90–95%) (Fig. 3D). The stimulatory effect of GA_3_ was not evident in the presence of 10^–4^ M ABA (ca. 3%). GA_3_ at10^−4^ M increased Timson´s index calculated for the coleorhiza and radicle by the factor of 24 and 27, respectively (Table 1B). When used together with ABA, GA_3_ resulted in a Timson’s index increase by the factor of 34 and 20 in the coleorhiza and radicle, respectively.

### Effects of KAR_1_ on GA_s_ contents in embryos from germinated caryopses

**Fig. 4:**
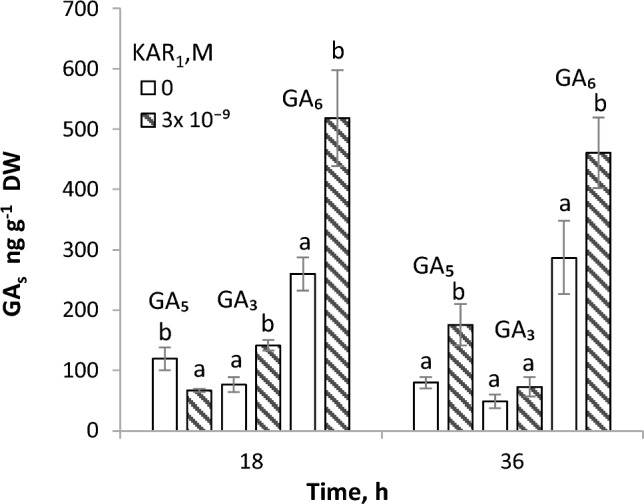
Effects of KAR_1_ on the GA_s_ contents in embryos of *A. fatua* caryopses after 18 and 36 h of germination. After 36 h, coleorhiza emerged in 49% of caryopses. Vertical bars indicate ± SD. One way ANOVA with Duncan´s post hoc test was used to test for significance of differences. Means denoted by different letters differ significantly (*P* < 0.05, *n* = 3)

Contents of GA_s_ from non-13-hydroxylation, GA_4_ and GA_7_, and 13-hydroxylation pathway, GA_1_, GA_5_, GA_3_ and GA_6_ in embryos isolated from caryopses germinated for various periods of time in water or KAR_1_ solutions was determined (Fig. 4). After 18 h of germination, KAR_1_ did not affect the contents of GA_1_, GA_4_ and GA_7_ (not shown) and decreased the GA_5_ content, whereas the contents of GA_3_ and GA_6_ increased (ca. 2 times). When the period of germination in the presence of KAR_1_ was extended to 36 h, the GA_5_ and GA_6_ contents in embryos were higher than in embryos from caryopses germinated in water; the increase was 1.5–2.2 times.

### Effects of PAC, KAR_1_ and PAC + KAR_1_ on radicle emergence as well as ABA and GA_s_ contents and ABA/GA_s_ ratio in embryos

**Fig. 5:**
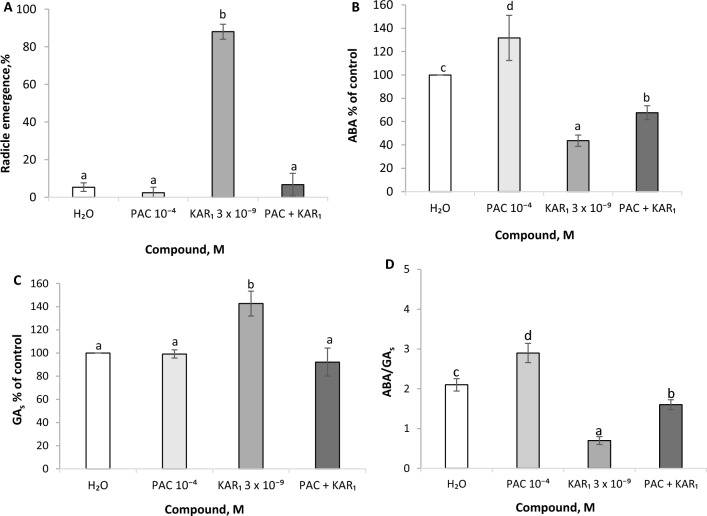
Effects of KAR_1_, PAC and KAR_1_ + PAC on the final radicle emergence of *A. fatua* caryopses (**A**) as well as on GA_s_ (GA_5_, GA_3_, GA_6_) (**C**) and ABA (**B**) contents in embryos after 30 h of caryopsis germination (presented as % of control content) and the ABA/GA_s_ ratios (**D**) in embryos. One way ANOVA with Duncan´s post hoc test was used to test for significance of differences. Means denoted by different letters differ significantly (*P* < 0.05, *n* = 3)

Radicles of dormant caryopses were almost unable to emerge when kept in water or in PAC solutions; ca. 2–5% of the radicles emerged (Fig. 5A). In contrast, KAR_1_-treated caryopses germinated nearly completely (ca. 90% showed emerged radicles). PAC applied in combination with KAR_1_ almost totally counteracted the stimulatory effect of the latter; ca. 6% of the caryopses showed emerging radicles. Contents of ABA, GA_5_, GA_3_ and GA_6_ (the 13-hydroxylation pathway) were determined in embryos isolated from caryopses germinated for 30 h in water, in the presence of PAC, KAR_1_ and the combination of PAC and KAR_1_. PAC was found to enhance, by 30%, the ABA content compared to embryos from water-germinated caryopses (Fig. 5B). In contrast, KAR_1_ applied alone decreased (by 60%) the ABA content. When PAC was applied simultaneously with KAR_1_, the ABA level was lower (by 30%) than that in embryos from water-germinated caryopses, but was higher than that in KAR_1_-treated ones. PAC did not affect the GA_s_ content, whereas KAR_1_ increased it by 40% (Fig. 5C). The GA_s_ content was similar in embryos from caryopses incubated in water, in the presence of PAC or KAR_1_ applied simultaneously with PAC. The ABA/GA_s_ ratios showed large treatment-dependent differences; the ratio of 1.6 to 2.9 was associated with the absence of germination, the ratio of 0.6 being characteristic of germination resulting from KAR_1_ treatment (Fig. 5D).

### Effects of KAR_1_ on the GA_s_ content and ABA/GA_s_ ratio in coleorhiza and radicle

**Fig. 6:**
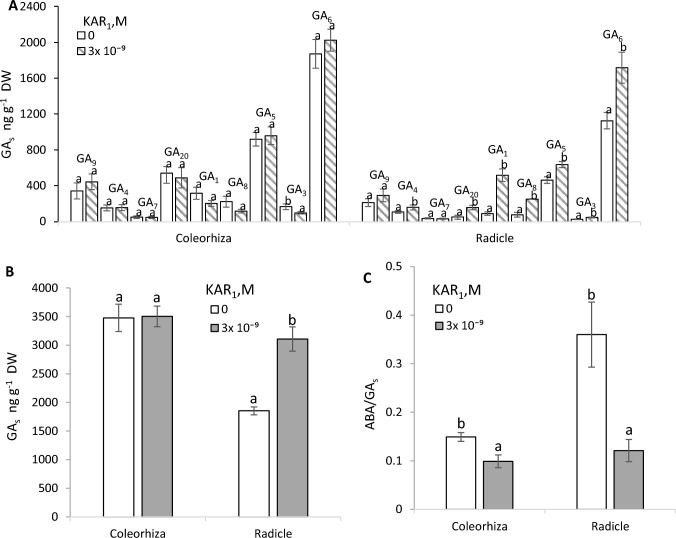
Effects of KAR_1_ on the GA_s_ (GA_9_, GA_4_, GA_7_, GA_20_, GA_1_, GA_8_, GA_5_) (**A**) contents, total GA_s_ content (**B**) and the ABA/GA_s_ ratios (**C**) in coleorhiza and radicle after 24 h germination of *A. fatua* caryopses. One way ANOVA with Duncan´s post hoc test was used to test for significance of differences. Means denoted by different letters differ significantly (*P* < 0.05, *n* = 3)

KAR_1_ did not affect the GA_9_, GA_4_, GA_7_, GA_20_, GA_1_, GA_8_, GA_5_ and GA_6_ contents in the coleorhiza of caryopses germinated for 24 h, but decreased the GA_3_ content (Fig. 6A). The content of all GA_s_ except for GA_9_ and GA_7_ was increased in radicles of KAR_1_-treated caryopses, the highest effect being seen in GA_1_; the GA_1_ content increased by the factor of ca. 6. The total content of all the bioactive GA_s_ from both GA_s_ biosynthesis pathways was similar in the coleorhiza from untreated and KAR_1_-treated caryopses (Fig. 6B). When caryopses were treated with KAR_1_, the bioactive GA_s_ content in the radicles was 1.7 times higher than that in the radicles of untreated caryopses. The ABA/GA_s_ ratios in the coleorhiza and radicles showed KAR_1_ to decrease the ratio by the factor 1.5 and 3, respectively (Fig. 6C).

## Discussion

### Relationship between exogenous KAR_1_ or GA_3_ and exogenous ABA

The results presented confirm the findings, reported by previous studies, that KAR_1_ applied at very low concentrations is able to remove dormancy in caryopses of *A. fatua* (Kępczyński 2018, 2023; Fig. 3), thus enabling almost all the caryopses to complete germination. The time course of germination in grasses, e.g. *Brachypodium distachyon* (González-Calle et al. 2015) and *A. fatua* (Kępczyński et al. 2021) involves two stages: the coleorhiza emergence (CE) at the first stage and the radicle emergence (RE) at the second, either stage or the two combined being used as a criterion for caryopsis germination (Gubler et al. 2008; Gendreau et al. 2008; Jacobsen et al. 2013; González-Calle et al. 2015). The coleorhiza is recognized as being mainly responsible for dormancy control in caryopses of barley (Barrero et al. 2009), *B. distachyon* (González-Calle et al. 2015) and *A. fatua* (Holloway et al. 2020; Kępczyński et al. 2021), and plays a role similar to that of the endosperm in dicot seeds. Radicle emergence through the endosperm or coleorhiza is regarded as a completed germination (Bewley et al. 2013). KAR_1_ turned out to be very effective in inducing CE and RE of dormant caryopses, not only when applied alone (Kępczyński et al. 2021; Fig. 3A, B), but also in the presence of ABA (Fig. 3A, B), a compound which inhibits germination of non-dormant *A. fatua* caryopses (Kępczyński et al. 2021). However, in an experiment with *Arabidopsis* seeds, KAR_1_ was unable to overcome ABA inhibition of seed germination (Nelson et al. 2009). Like in previous studies, GA_3_, similarly to KAR_1_, released dormancy in *A. fatua* caryopses, but much higher concentrations were needed (Kępczyński 2018; Fig. 3C, D). This is consistent with findings from other seeds, e.g. those of *Arabidopsis* and *Brassica tournefortii*, more sensitive to KAR_1_ than to gibberellin (Daws et al. 2007; Stevens et al. 2007; Nelson et al. 2009). In the presence of ABA, a higher concentration of GA_3_ than KAR_1_ was required for dormant *A. fatua* caryopses to germinate (Fig. 3). Antagonistic effects of both KAR_1_ and GA_3_ towards ABA were more evident in CE than in RE. This indicates that, in the presence of ABA, the coleorhiza is more sensitive to KAR_1_ and GA_3_ than the radicle, which is consistent with previous results showing a stronger response of dormant caryopses to KAR_1_ in the coleorhiza than in the radicle (Kępczyński et al. 2021). Not only did both, KAR_1_ and GA_3_, increase the percentage germination in the presence of ABA, but they also accelerated caryopsis germination, even when the final germination was not affected (Table 1). Taking into account the final germination and the germination speed, it can be concluded that KAR_1_ and GA_3_ reduced the sensitivity of both the coleorhiza and radicle to ABA; however, the effect was more effective in the coleorhiza. It was demonstrated earlier (Kępczyński et al. 2023) that NO, another dormancy-releasing agent, was able to reduce the sensitivity of *A. fatua* caryopses to ABA.

### The relationship between exogenous KAR_1_ and endogenous GA_s_ and ABA

#### Embryos

*A. fatua* embryos showed the presence of GA_s_ from the non-13-hydroxylation and 13-hydroxylation pathways ( Kępczyński et al. 2021; Fig. 4), recognized as bioactive (Hedden 2016). The dormancy releasing effect of KAR_1_ was associated only with an increase in the 13-hydroxylation pathway GA_s_ (Fig. 4), in contrast to the dormancy releasing effect of NO, which did not affect the contents of GA_s_ from both pathways in the embryos (Kępczyński et al. 2023). This suggests a difference between these compounds in the dormancy release mechanisms. The stimulating effect of KAR_1_ and NO on germination of dormant caryopses was strongly counteracted by PAC (Kępczyński 2018; Ruduś et al. 2019; Kępczyński et al. 2023; Fig. 5A) regarded as a GA_s_ biosynthesis inhibitor (Desta and Amare 2021), which suggests a requirement for endogenous GA_s_ in response to both compounds. PAC applied alone to caryopses was reported to not affect GA_s_ content in embryos (Kępczyński et al. 2023; Fig. 5C), nor did it affect the GA_s_ content when caryopses were treated with NO (Kępczyński et al. 2023). However, PAC reduced the GA_s_ content when applied simultaneously with KAR_1_, the decline being associated with a reduced stimulatory effect of KAR_1_ on germination of dormant caryopses (Fig. 5A, C). It was also reported earlier that PAC, in addition to influencing the GA_s_ content, can increase the content of ABA by increasing its synthesis and/or inhibiting its catabolism (Yamaguchi et al. 2007; Desta and Amare 2021). The ABA content, which was reduced by KAR_1_ (Kępczyński et al. 2021; Fig. 5B) as a result of degradation to phaseic acid (Kępczyński 2023), and by NO (Kępczyński et al. 2023), was increased due to PAC being used alone (Kępczyński et al. 2023) or simultaneously with KAR_1_ (Fig. 5B). Taken together, this might confirm different mechanisms involved in dormancy release in *A. fatua* caryopses by KAR and NO.

It is widely accepted that the ABA/GA_s_ ratio is mainly responsible for the dormancy level and seed germination (Rodriguez et al. 2015; Tuan et al. 2018). A high ABA/GA_s_ ratio associated with a high ABA and low GA_s_ contents is responsible for dormancy. A low ratio, associated with low ABA and high GA_s_ levels, allows germination. ABA plays a crucial role in the induction and maintenance of seed dormancy (Rodriguez-Gacio et al. 2009). GA_s_ act antagonistically to ABA, promote dormancy release and are required for germination (Kucera et al. 2005; Bewley et al. 2013). Taking into account the ABA/GA_s_ ratio in *A. fatua* embryos, it can be seen that the KAR_1_-effected dormancy release is associated with a marked decrease in the ratio (Fig. 5D). Its high level in embryos from caryopses germinated in water or in the presence of PAC or PAC + KAR_1_ is presumably responsible for inability of the caryopses to transit from dormancy to germination. Reduction of the ABA/GA_s_ ratio by KAR_1_ involves a decrease in the ABA content and a simultaneous increase in GA_s_ contents (Fig. 5B, C), while in the case of NO the effect is associated only with a decrease in the ABA content (Kępczyński et al. 2023). Interestingly, the ABA levels in *Arabidopsis* seeds were not affected by KAR_1_ prior to radicle emergence, although KAR_1_ did effectively remove dormancy (Nelson et al. 2009). Moreover, KAR_1_ only slightly increased the GA_4_ level. It has also been shown that KAR_1_ can delay germination of non-dormant soybean seeds by a change in the ABA/GA_4_ ratio due to the biosynthesis of ABA and GA_4_ being enhanced and impaired, respectively (Meng et al. 2016).

#### Coleorhiza

Since the coleorhiza is considered to be responsible for the dormancy state of barley (Millar et al. 2006; Barrero et al. 2009) and *A. fatua* (Holloway et al. 2020) caryopses, GA_s_ from the non-13-hydroxylation and 13-hydroxylation pathways were determined not only in embryos, but also in the coleorhiza. KAR_1_ was found to not increase the contents of bioactive GA_s_ from both pathways (Fig. 6A, B). Likewise, the GA_9_, substrate for GA_4_ and GA_7_, as well as GA_20_, the substrate for GA_1_ and GA_5_, were not affected by KAR_1_. KAR_1_ was earlier demonstrated to be capable of reducing the ABA content in the coleorhiza (Kępczyński et al. 2021). Taking these results into account, it can be concluded that the KAR_1_-effected control of the ABA but not GA_s_ contents in the coleorhiza plays a vital role in releasing caryopsis dormancy. It has been previously postulated that endogenous GA_s_ in the coleorhiza are not involved in dormancy release of barley caryopses since expression of the genes involved in the GA_s_ synthesis (*KAURENOIC ACID OXIDASE1*) was upregulated only in the coleorhiza of dormant caryopses, and expression of *GIBBERELLIN 2-OXIDASE1* responsible for GA_s_ inactivation was upregulated in coleorhiza of non-dormant caryopses (Barrero et al.^2009^). Thus, ABA in the coleorhiza plays an essential role in the control of caryopsis dormancy in barley (Barrero et al. 2009) and *A. fatua* (Holloway et al. 2020; Kępczyński et al. 2021), GA_s_ not being involved. The ABA/GA_s_ ratio showed that, like in the embryos, KAR_1_ reduces the ratio in the coleorhiza (Fig. 6C), but in contrast to embryos the effect is associated only with a reduction in the ABA content, which probably could allow an increase in the activity of enzymes responsible for weakening the coleorhiza, as was postulated for barley (Barrero et al. 2009), *Brachypodium distachyon* (Gonzalez-Calle et al. 2015) and *A. fatua* (Holloway et al. 2020).

#### Radicle

KAR_1_ increased the total content of bioactive GA_s_ from both pathways (Fig. 6B), which was related to an increase in the content of four gibberellins (GA_1_, GA_5_, GA_3_ and GA_6_) from the 13-hydroxylation pathway and one, GA_4_, from the non-13-hydroxylation pathway (Fig. 6A). The largest impact of KAR_1_ was recorded in GA_1_ the content of which was increased by ca. 470%, suggesting that it is mainly GA_1_ that is required for radicle growth. The contents of GA_4_, GA_5_, GA_3_ and GA_6_ increased by 50–70%. Moreover, GA_20_—a substrate for GA_1_ biosynthesis—was markedly, by ca. 40%, increased by KAR_1,_ which may suggest the potential to synthesize GA_1_, enabling a further increase of its content and, perhaps, also the remaining GA_s_ of this pathway. The increase of the content of GA_8_, a GA_1_ catabolite, by KAR_1_ may indicate the regulation of the bioactive gibberellin concentrations also through deactivation. The presented data could indicate that GA_s_ from the 13-hydroxylation pathway play the main role in radicle growth; GA_4_ from the non-13-hydroxylation pathway seems to be less important (Fig. 6A). In contrast to KAR_1_ effect on the GA_s_ content, KAR_1_ reduced the content of ABA (Kępczyński et al. 2021), a GA_s_ antagonist, considered as a positive regulator of dormancy and negatively affecting seed germination (Hilhorst 1995). Thus, the balance between ABA and GA_s_ was altered by KAR_1_, like in the coleorhiza. It is worth emphasizing that the reduction of the ABA/GA_s_ ratio by KAR_1_ in the coleorhiza is associated only with a reduction in the ABA content, whereas in the radicle, in addition to the reduction of the ABA content, there is also an increase in the GA_s_ content. Considering the antagonistic effects of ABA and GA_s_, a change in the ABA/GA_s_ ratio possibly enables the radicle to grow and break through the coleorhiza, allowing germination to be completed. Thus, KAR_1_-associated dormancy release, involves—in addition to a reduction of the ABA/GA_s_ ratio in the coleorhiza—probably also an increase of the radicle’s expansive force by a decrease of the ABA/GA_s_ ratio, making it easier for the radicle to penetrate the coleorhiza. It was earlier proposed that RE may depend not only on weakening of the coleorhiza, but also on the expansive force of the radicle (Barrero et al. 2009). Our previous study (Kępczyński et al. 2021) allowed to suggest that the inability of dormant caryopses to complete germination, probably mainly due to the endogenous ABA concentration being too high, might involve inhibition of the cell-cycle activation.

To summarize, like in previous studies (Kępczyński 2018, 2023), KAR_1_ and GA_3_, very actively induced dormancy release in *A. fatua* caryopses. Both compounds reduced the sensitivity of the coleorhiza and radicle to exogenous ABA. The dormancy releasing KAR_1_ effect was associated with a decrease in the ABA/GA_s_ ratios in embryos, coleorhiza and radicle before germination was completed. The mechanism of dormancy release by KAR_1_ is related to a reduction of the coleorhiza sensitivity to ABA and a decrease in the ABA/GA_s_ ratios in the coleorhiza, regarded as playing a key role in maintaining caryopsis dormancy, by a decrease in the ABA level. A KAR_1_-induced reduction of the radicle ABA/GA_s_ ratio, involving a large increase in the content of bioactive GA_s_, particularly GA_1_, as well as a reduction of the ABA level are probably required for the radicle to grow and break through the coleorhiza. Thus, caryopsis dormancy release under the influence of KAR_1_ requires a reduction of the ABA/GA_s_ ratio in the coleorhiza and also in the radicle, which probably allows the radicle expansion force to increase, thus facilitating the coleorhiza puncture. It has been shown for the first time here that a reduction of the ABA/GA_s_ ratio is necessary for dormancy release by KAR_1_. The results presented may also indicate that the mode of KAR_1_-effected dormancy release differs between monocot and dicot seeds.

## Data Availability

The data sets generated and/or analysed during the current study are available from the corresponding author on a reasonable request.

## Abbreviations

CE: Coleorhiza emergence
GA_s_: Gibberellins
KAR_1_: Karrikin 1
NO: Nitric oxide
PAC: Paclobutrazol
RE: Radicle emergence
